# Real-Time Early Warning System Design for Pluvial Flash Floods—A Review

**DOI:** 10.3390/s18072255

**Published:** 2018-07-12

**Authors:** Melisa Acosta-Coll, Francisco Ballester-Merelo, Marcos Martinez-Peiró, Emiro De la Hoz-Franco

**Affiliations:** 1 Department of Electronic Engineering, Universitat Politècnica de València, 46022 València, Spain; fballest@eln.upv.es (F.B.-M.); mpeiro@eln.upv.es (M.M.-P.); 2 Department of Computer Sciences and Electronic, Universidad de la Costa, Barranquilla 080020, Colombia; edelahoz@cuc.edu.co

**Keywords:** pluvial flooding, urban drainage, flash floods, early warning system, flood risk assessment, real-time

## Abstract

Pluvial flash floods in urban areas are becoming increasingly frequent due to climate change and human actions, negatively impacting the life, work, production and infrastructure of a population. Pluvial flooding occurs when intense rainfall overflows the limits of urban drainage and water accumulation causes hazardous flash floods. Although flash floods are hard to predict given their rapid formation, Early Warning Systems (EWS) are used to minimize casualties. We performed a systematic review to define the basic structure of an EWS for rain flash floods. The structure of the review is as follows: first, Section 2 describes the most important factors that affect the intensity of pluvial flash floods during rainfall events. Section 3 defines the key elements and actors involved in an effective EWS. Section 4 reviews different EWS architectures for pluvial flash floods implemented worldwide. It was identified that the reviewed projects did not follow guidelines to design early warning systems, neglecting important aspects that must be taken into account in their implementation. Therefore, this manuscript proposes a basic structure for an effective EWS for pluvial flash floods that guarantees the forecasting process and alerts dissemination during rainfall events.

## 1. Introduction

Flooding is considered as one of the major threats to human civilization and is directly attributed to heavy precipitation leading to loss of life, infrastructure damage, as well as huge economic losses [1,2]. Climate change, intense natural resource exploitation and inappropriate land use have altered the hydrological response of catchments. These factors increase the frequency and magnitude of floods. Similarly, a combination of an exposed, vulnerable and ill-prepared population may exacerbate such situations and even generate additional risks. The insufficient capacity of public authorities and rescue services to act diligently in these situations increases later mentioned risks [3,4,5]. 

Cities with high population density present a higher disaster risk. They are expected to experience the effects of climate change with the increment of intensity and frequency of harmful events such as flash floods [6]. Vulnerability to disasters in urban areas is a combination of interrelated physical, sociocultural, economic, and institutional conditions [7]. 

Different types of floods can affect urban areas and some of them may be more applicable to some regions than others. These floods are mainly classified into four types: coastal, fluvial, pluvial and flash floods.

Coastal flooding results from a combination of extreme climatic phenomena. The sea level exceeds the elevation of the land or of a natural or human barrier; water flows and floods the land behind it [5,8]. When the coasts are constantly exposed to large waves, the natural and human-engineered barriers break down, increasing the risk of flooding. Also, this flood can be caused by earthquakes, submarine volcanic eruptions, subsidence and coastal erosion [9].

The flooding that affects the vast majority of the world’s regions is fluvial flooding or river flooding. This type of flood occurs when the rivers overflow or burst their banks due to excessive rainfall over an extended period of time and spill onto the floodplain [10,11]. It can also be caused by rapid snowmelt and ice jams and occurs in any size channel from small streams to huge rivers. 

Pluvial flooding or surface water flooding is a problem in many cities and occurs when, during high intensity rainfall, the sewage and drainage system becomes overwhelmed and excess water cannot be absorbed into the soil [12]. This problem is enhanced in cities with insufficient or non-existent sewer systems. Although fluvial floods are more devastating than pluvial flooding, they do not occur that often. Pluvial floods come with less damage but, the frequency is higher and the cumulative damage over the years can be just as high as with fluvial flooding events [13,14].

Of all the negative impact generated by floods, none is as harmful as flash floods (based upon the ratio of fatalities to people affected), which cause millions of dollars in property damage every year [15]. The World Meteorological Organization (WMO) defines flash floods as “*a flood of short duration with a relatively high peak discharge*” [16]. The American Meteorological Society states: “*a flash is a flood that rises and falls quite rapidly with little or no advance warning, usually as the result of intense rainfall over a relatively small area*” [6]. The U.S. National Weather Service describes them as: “*a rapid and extreme flow of high water into a normally dry area, or a rapid water level rise in a stream or creek above a predetermined flood level, beginning within six hours of the causative event (e.g., intense rainfall, dam failure, ice jam)*” [7]. These floods are typically caused by coastal, fluvial and pluvial systems and convective thunderstorms as well as extreme events such as hurricanes, severe thunderstorm, tropical storms or tsunami [15]. Dam break, a levee break and snow melting in rivers during winter and spring months can result in flash floods.

As reported by The World Bank, in 2008 half of the world’s population lived in urban areas but in 2030 this number will increase to 60% and 70% in 2050 [9]. This accelerated urbanization compounds flood risk since, in most cases, it is done in an unplanned way [17]. Therefore, urban flood disaster prevention and mitigation are a recognized international priority that includes assessing flood hazards and risks and preparing effective flood mitigation measures [16,18]. 

**Figure 1:**
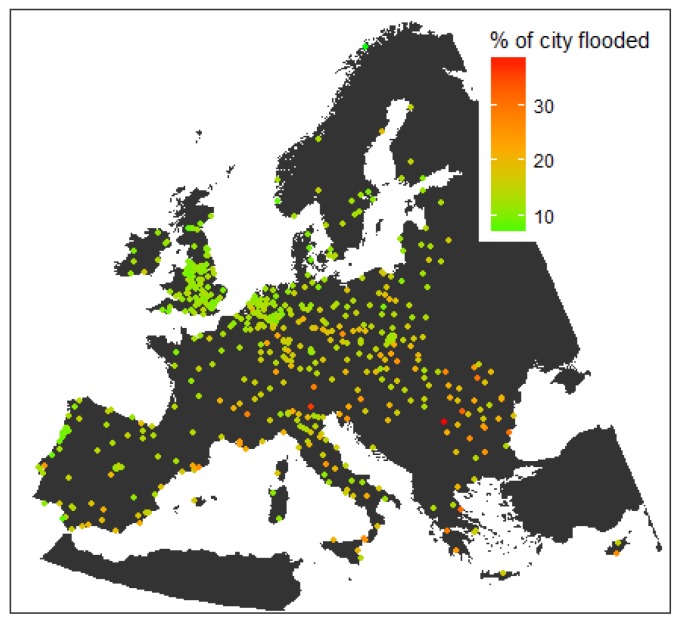
Pluvial flood impact in European cities [20].

Although, historically, the most reported flood events are fluvial followed by pluvial and sea water, pluvial floods have increased in cities and have the highest proportion of occurrence since 2000 compared to other types of floods in the same period [19]. Figure 1 shows a preliminary assessment of pluvial flood impacts for 571 cities across the continent of Europe developed by Guerreiro et al. [20] using emerging global datasets and cloud computing.

The United Kingdom is one of the areas in Europe most affected by pluvial flooding. Around 5% of the urban population is exposed to an annual pluvial flood risk of 0.5% or greater [21]. It is estimated that, by 2050, 3.2 million people in urban areas could be at risk from pluvial flooding [21].

Not only European cities are affected by pluvial flooding. In Japan, approximately USD 1 billion in damage occurs annually due to pluvial floods affecting densely populated urban areas with poor drainage systems [22]. However, these pluvial events were recorded not only during heavy rainfall but also during moderate to low rainfall events.

Due to the deficient drainage infrastructure in the cities, these pluvial floods turn into dangerous flash floods and not only affect the economy but also lead to human losses as is the case for the city of Barranquilla (Colombia). In this city, during high rainfall events, the streets become torrential streams endangering pedestrians and drivers. Since there is no system that alerts in a timely manner the community about the danger of these floods, pedestrians and drivers trying to cross the streets are washed away by the dangerous streams [23].

Chinese cities including Beijing, Shanghai, Guangzhou, Shenzhen, Nanjing, and Hangzhou are also affected by pluvial flash floods [24]. In July 2012, a pluvial flash flood event caused by road inundations was registered in Beijing and claimed 79 lives [25].

Pluvial flash floods are not simply caused by weather phenomena. They depend not only on the amount and duration of precipitation but also on the hydrological characteristics of the basin such as runoff magnitude, antecedent moisture condition, drainage area, soil type and land [26,27]. Hydraulic parameters, such as water level and water velocity, are variables that are involved in the loss of stability of people and vehicles during urban flash floods [23], and it is necessary to measure and monitor these parameters in real time. If, during rain events, water level and speed exceed safety levels, it is necessary for the alarm system to warn the community about the imminent danger.

Although there is a huge demand for understanding pluvial flash floods in cities, until now, few works have attempted to systematically examine the potential impacts of a pluvial flash flood in cities in order to develop efficient solutions [28]. 

In order to mitigate the risk of human losses and economic damages caused by floods in cities, measures of adaptation and minimization should be considered. The International Strategy for Disaster Reduction (ISDR) has classified these measures into structural and non-structural [29,30]:

(a) Structural measures

They include the construction of physical structures to reduce or avoid potential impacts of hazards such as protection, retention and drainage systems, as well as the use of engineering techniques to improve resistance and community resilience [29]. Most of these measures involve a high investment of economic resources and implementation time is medium- to long-term.

(b) Non-structural measures

These actions do not involve building physical structures, but rather use existing knowledge, laws or policies to reduce risk and its impacts [29,31]. These measures are classified as passive and active. Active nonstructural measures are those that promote direct interaction with people, such as training, local management, early warning systems (EWS) for people, public information, among others. Non-structural passive measures involve policies, building codes and standards, and land use regulations.

Early warning systems are nonstructural tools useful to populations that do not have sufficient resources to minimize the risk of flooding. They are tools to reduce economic losses, and protect the life and property of a community [32]. Information sent by the EWS allows people to take action before the disaster takes place. Recent studies demonstrate that these systems have significant benefits that greatly exceed their costs [33].

The aim of this manuscript is to provide guidelines to develop an effective EWS for pluvial flash floods in real time. Understanding the hazard posed by pluvial flash floods in cities and the limited information available for EWS design to mitigate this risk is mandatory. The causes and variables that influence the formation of flash floods in urban areas, as well as the key elements of an EWS, are described. Likewise, different EWS for pluvial flash floods implemented worldwide were reviewed to determine the primary and secondary instruments used to measure the variables and the methods for processing information and alerting the community at risk.

Each architecture describes the instruments and methods used for the detection, monitoring and real-time analysis of variables related to flash floods and alert dissemination. From the reviewed projects, the most used instruments to measure hydrological and hydraulic variables during pluvial flash floods were selected. Also studied were the communication protocols to send the information and main media for alert dissemination. 

In this review, we identified the need for forecasting and dissemination–communication processes to have fail-safe systems. These processes guarantee that the community receives timely alerts. However, none of the reviewed projects had a fail-safe system for these processes. The lack of these systems makes early warning systems more susceptible to the loss of measured data. Therefore, alerts cannot be sent timely to the community. 

For this reason, this manuscript proposes a basic structure for an effective pluvial flash flood early warning system that guarantees the dissemination and communication of alerts during rainfall events. This proposed EWS suggests which hydrological and hydraulic variables should be monitored in real-time during rainfall events; compares the characteristics of communication protocols and the most effective media to disseminate the alerts.

## 2. Pluvial Flash Flood Intensity

Developing techniques and criteria for solving the complex problems associated with runoff in urban areas is one of the most challenging tasks for hydrologic engineers. During high-intensity rainfall events, drainage systems become rapidly saturated. For this reason, soil cannot absorb the water quickly enough. There are a number of factors that affect the intensity of pluvial flash floods. Climate change, extensive and rapid urbanization and unsustainable urban development combined with management failure are considered the main factors for the development of pluvial flash floods. In general, higher precipitation intensity can result in an additional runoff. A deficient drainage system also increases the volume [6]. Likewise, there are other factors involved with runoff production processes such as soil characteristics, land cover, land use and basin conditions that increase the runoff intensity. This section briefly describes how climate change effects, urbanization, soil and basin characteristics increase the runoff intensity in urban areas.

### 2.1. Climate Change

A number of studies and reviews have assessed the influence of a combination of climate change and rapid urban development in flood risk [34,35,36]. In recent years, in China, the increment of high intensity and short duration rainfalls has been observed. Also, changes in the upper extremes of the distributions of high volume precipitation indexes have occurred in a broader area [37,38,39]. The increase of precipitation frequency and intensity aggravates the problem of pluvial flooding, representing a changing biophysical condition for urban areas [13].

### 2.2. Urbanization

Urbanization and vegetation area reductions are significant threats that affect water quality and increase the risk of flooding in urban areas. In the UK alone, over 80% of the population live in urban areas and the population has risen from 32 million in 1901 to 64.6 million in 2014 [40]. During the period 1990–2015, China’s urban population increased from 302 million to 771 million, implying an average annual growth rate of about 6.2%. Over the same period, China’s urbanized population had an average annual growth rate of 12% that was near twice the average annual growth rate of the overall population [41]

Urban densification, deforestation and inadequate urban drainage design have led to greater runoff volumes since the percentage of impermeable surfaces and compacted soils is greater [42]. Likewise, road grids, alterations to the natural vegetation, and sometimes channelization of streams have produced faster runoff [43]. 

### 2.3. Soil Characteristics

In urban areas, soil moisture and soil permeability are critical properties that influence the formation of flash floods. Soil moisture is considered the most important soil factor for rapid runoff and flash flooding because it can vary significantly, even on a sub-daily time scale [44]. In dry conditions, each soil has a specific rate at which it can absorb rainfall, called the infiltration capacity. When the rainfall rate exceeds the infiltration capacity, runoff will occur [6]. If the soils are saturated due to previous moisture conditions, the infiltration capacity is lower, resulting in higher runoff. 

The infiltration capacity can also be affected by the permeability of the soil. This property depends on the different characteristics of the soil, such as texture, crust formation, soil compaction, soil contraction and expansion, microbial activity, soil hydraulic conductivity and root distribution. Soil texture is the most important [6,45] and indicates percentages of different grain sizes such as sand, silt and clay in the soil. Sandy soils have greater infiltration rates than clay and silt soils because sand particles are larger and more separated.

### 2.4. Basin Characteristics

The physical properties of a basin-like size, shape and surface roughness and its streams, influence the amount and the timing of runoff [6]. The size and shape of the basin directly influence the total volume of runoff that drains from that basin. In large basins, the runoff will take longer to reach its outlet than small basins because of the long distance to travel. In addition, rainfall events over larger basins will probably impact only a portion, but it could cover the entire small basin. 

The relation between the infiltration rate and the basin’s slope is inversely proportional. If the basin’s slope increases, the infiltration rate decreases. The reason is that gravity pulls less water into the land surface and more water across that surface [43]. Unlike the basin’s slope, surface roughness has a directly proportional relation with infiltration rate; reducing channel roughness causes less infiltration and faster streamflow velocities [6].

## 3. Early Warning System Basic Architecture

This section describes the architecture of an early warning system for the dissemination of timely alerts during pluvial flash floods. EWS is understood as a set of procedures, steps or key elements related and interconnected with each other [46]. The United Nations has defined early warning systems as “An integrated system of hazard monitoring, forecasting and prediction, disaster risk assessment, communication and preparedness activities systems and processes that enable individuals, communities, governments, businesses and others to take timely action to reduce disaster risks in advance of hazardous events” [29]. According to the World Meteorological Organization (WMO) and International Strategy for Disaster Reduction (ISDR) [4,16] the architecture of an effective EWS is divided into the following key elements or structures: Disaster risk knowledge, Forecasting, Dissemination–Communication and Preparedness–Response. 

(a) Disaster Risk Knowledge

An event becomes a disaster when it abruptly affects a community’s daily activities and involves human and material losses and has economic or environmental impacts [46]. It would also be considered as a disaster when damage exceeds the community’s ability to respond with their own resources [18]. When there is a greater knowledge of the risk to which a population is exposed, this leads to the improvement of the processes of risk management, reduction and adaptation [47,48]. The knowledge contains information that can be used to make decisions and actions that allow the community to improve their capacity to react to disaster risk in a timely manner [49,50].

In this key element, it is necessary to identify hazards, the exposure, vulnerabilities and risks of a population. According to the United Nations Office for Disaster Risk Reduction (UNISDR) [29], a hazard is any substance, phenomenon or situation that affects a community and has the potential to damage people and their property. The hazards can be classified into natural, biological, technological and societal. Hazard identification implies what might happen and where.

Vulnerability relates to a number of factors such as physical, economic, social and environmental [51]. The Asian Disaster Preparedness Center defines the concept of *vulnerability* as “the factors or constraints of an economic, social, physical or geographic nature, which reduce the ability to prepare for and cope with the impact of hazards” [52]. The UNISDR defined *vulnerability* as “the conditions determined by physical, social, economic and environmental factors or processes which increase the susceptibility of an individual, a community, assets or systems to the impacts of hazards” [29]. 

The exposure is “The situation of people, infrastructure, housing, production capacities and other tangible human assets located in hazard-prone areas” [29]. 

The risk is “the probability that negative consequences may arise when hazards interact with vulnerable areas, people, property, environment” [52]. 

To reduce the risk of flooding in urban areas, the data collected should be relevant and concise, qualitative or quantitative, and should be obtained through official sources [16,53]. The following areas should be covered:

- Historical background
- Geographical aspects
- Environmental and physical aspects
- Socio-cultural aspects
- Economic aspects

Likewise, vulnerability assessment of the area at risk is necessary. The various components and essential functions of a city that may be at the heart of possible dangers should be considered [54]. Furthermore, the dynamic nature of hazards and vulnerabilities arising from processes such as urbanization, environmental degradation and climate change should be taken into account [16]. Developing a risk map allows the needs of the early warning system to be prioritized and preparations for disaster prevention and response to be guided [55].

The following questions must be answered:

- Are the hazards and the vulnerabilities well known?
- What are the patterns and trends in these factors?
- Are risk maps and data widely available?

To answer these questions, Fakhruddin et al. [56] propose an assessment methodology for flood risk by elaborating a map. The elaboration of the map is divided into two sections:

- First, the initial data processing, establishment of the hydrological model to predict runoff, probability analysis and elaboration of a flood risk map is performed.
- Second, interviews, discussion groups and workshops are conducted with the community at risk to determine vulnerability, taking into account community perceptions and historical records.

Finally, the result of these processes resulted in the development of an integrated system, flood risk map and response option. 

Once a detailed risk map is made and relevant information from the area is obtained, the warning design must be established. This process is complex and requires the integration of different activities, devices and the processing of large volumes of information. The Forecasting section describes the procedure for designing timely, clear and useful alerts for the community.

(b) Forecasting

For urban flash floods, the main goal of this key element is forecasting and establishing alert levels in real time. This process is divided into two sections: Monitoring and Information Processing. The Monitoring section monitors and transmits information on meteorological and hydraulic variables related to urban flash floods [57]. 

The Information Processing section receives the data of the meteorological and hydraulic variables, and through analysis tools, computer models and simulator design alert [58].

The forecasting process requires the use of a number of technologies and areas of expertise for the analysis of large volumes of data and predictions based on simulations. These technologies include sensors to measure meteorological and hydraulic variables and computational models and simulation software to process the information. It is necessary to provide an advanced visualization technology to interact with people at risk and a decision support system with remote access to assist public authorities and citizens in timely decision making [48,59].

According to the UNISDR [46], this section should answer the following questions:

- Are the right parameters being monitored?
- Is there a scientific basis for making forecasts?
- Can accurate and timely warnings be generated?

As part of the decision support tools during high rainfall events, different runoff and hydraulic models are available for urban flooding forecasting. They are a rainfall-runoff model and hydrodynamic models in 1D, 2D and 3D. Most of these models use as input the measurements of the amount of precipitation, water level and water velocity. Therefore, these variables should be monitoring in real time [60,61].

The alerts must be sent in a timely manner and the message transmitted must be clear and understandable for all people. The Dissemination–Communication section details the characteristics of alert messages during flash floods and the means used to send them.

(c) Dissemination–Communication

Sending and communicating warnings is the determining step between forecast and action [62]. Dissemination refers to sending the warning, while communication is achieved only when the information is received and understood [16]. Sending the alerts to people at risk during high-intensity precipitations is an extremely important phase in which the message should be simple and useful. This allows for adequate responses that help safeguard lives and livelihoods [55]. Dissemination and communication systems for alerts should be able to answer the following questions:

- Do warnings reach all those at risk?
- Are the risk and warnings understood?
- Is the warning information clear and usable?

To achieve positive answers to these questions, alerts must be available in different formats, such as text, graphics, colour coding, audio, etc. This facilitates the reception and action on warnings. According to the WMO [16], for alerts to be effective, their content should be brief, concise, understandable, and answer questions such as “What?”, “Where?”, “When?“, “Why?” and “How to respond?”. Also, detailed threat information using localized geographic references should be included. Dissemination of alerts must be done through multiple channels in order to reduce delays in delivery to end-users, as well as ensure it reaches as many people as possible. Channel failure should be prevented [4,53]. Likewise, credible sources, pre-identified and approved, should deliver warnings. Measures must be taken to promote trust among the public so that prompt action is taken once the message is received [63]. Some actions included are as follows:

- Dissemination of warnings through organizations or leaders
- Sending warnings through multiple credible sources
- Periodic and constant warnings
- Scientifically certified warnings

According to The Economist Intelligence Unit, Hong Kong is considered to be one of the safest cities in the world today, due to its ability to prepare and respond to disasters [54,56]. In 2015, they carried out a study to assess the preparedness of Hong Kong residents for community disasters and to identify factors that affect their behaviour during these events [31]. A total of 1023 residents aged 18 years or more were interviewed to answer a 19-item questionnaire, which evaluated the following aspects of disaster preparedness and response:

- Having information regarding their preparation
- A communication plan, evacuation strategies
- First-Aid and disaster knowledge
- Financial resilience
- Preparedness behaviours

Table 1 shows survey results regarding which sources people would use during a disaster. The study found that people over 65 were more likely to seek information through television and radio, while younger people responded more to social media.

Regarding which information they considered most important, the results show the following:

(1) Places to seek medical attention (92.2%)
(2) Evacuation routes (85.2%)
(3) Shelter information (84.8%)
(4) Details of the disaster (67.4%)
(5) Missing persons (65.2%)
(6) Victims (45.2%)

For the communication process, 65% of respondents would use emergency contact numbers on their mobile phones, but 73.1% had password-protected phones. In the event that the mobile phone network failed, 37.4% of the respondents stated that they would use a landline to communicate with their family, 32.5% said they would go home and 4% reported having a place to meet your family. This study concludes that, ultimately, most residents are interested in receiving additional information on disaster preparedness through the Internet using mobile devices and television.

EWS are considered effective not only when an alert is sent in a timely manner but when this alert is correctly understood and the community takes protective actions [64]. This implies active community participation in the design of EWS, as well as the preparation and response to the risk of flash floods.

(d) Preparedness–Response

Disaster preparedness includes all the activities necessary for a community to react to such an event [65]. It is necessary for the community to receive and correctly interpret issued alerts, so they may draw the necessary conclusions for actions to be taken, such as alerting local police or firefighters [64]. Many deaths have been recorded during flash floods worldwide, as people try to drive or walk across the streams of water unknowingly or poorly assessing the risk [63,66]. Therefore, it is necessary not only to issue flood warnings in a timely manner but also to identify the community’s perception of flash floods and the factors that influence their responses when receiving the warning.

The results of a public survey of 418 people in Boulder, Colorado, USA, on how people perceive, understand, and respond to flash floods and warnings received through different means were presented in 2015 [67]. They establish that people have different perceptions and concepts about flash floods and understandings of risk. The survey structure was divided into three main sections:

(1) Perceptions and understandings of flash flood risks
(2) Perceptions and interpretations of flash flood forecasts, warnings, and other alerts
(3) Protective decision making in response to flash flood warnings

In Section 3 of the survey, the ability of people to take protective measures during flash floods was examined. The survey mentions different types of warnings that could be sent during a flash flood and respondents answered what they will do if they heard the warning while driving, while in a building on the ground floor or below, or while outdoors. Table 2 shows the alert messages and the response actions of the respondents. The column on the left shows the warnings, the central column shows the percentage of respondents coded in that category, and the column on the right shows the responses obtained in this survey.

It is necessary not only to notify people about the danger of a sudden flood but also to motivate them to take protective measures. According to Quevauiller and Innocenti [68], the following recommendations could improve people’s response:

- Institutional and social conditions that must be fulfilled to ensure timely decision-making regarding the warnings should be as follows:
  1. Alert dissemination and communication
  2. Clarity regarding responsibilities in case of warning
  3. Preparing authorities and communities to respond to the disaster
- The involvement of local communities and authorities in the design of EWS increases the effectiveness of the entire early warning process and thus leads to a greater and better response to an alert.

According to the ISDR, each key element has key actors that should be involved to develop a people-centred Early Warning System. Table 3 shows the key actors for each key element.

Another feature of the effectiveness of an EWS is that the key elements must be interrelated. The activities carried out in each section should be aimed at the satisfactory development of the following section.

## 4. Real-Time EWS for Pluvial Flash Floods

This section presents different architectures of early warning systems for pluvial flash floods implemented worldwide. Each project installed different types of sensors to monitor variables used in urban flood forecasting and modelling.

Wireless communication was the most used technology for transmitting data to the processing centre. On the other hand, each one developed a different method for information processing and alert dissemination.

### 4.1. Florida, United States

**Figure 2:**
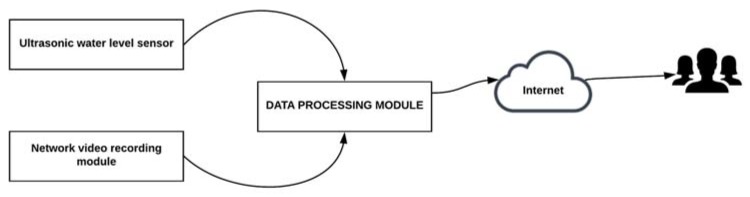
General structure of motes-based sensor network for the Florida (United States) project.

Chang and Guo [69] proposed a motes-based sensor network for water level monitoring and real-time video delivery of channel status. This system consists of three modules: Ultrasonic Water Level Monitoring Module, the Network Video Recording Module and Data Processing Module. All modules are connected to a photovoltaic system for power supply. Figure 2 illustrates a general structure of motes-based sensor network for the Florida (United States) project.

- The *Ultrasonic Water Level Monitoring module* uses an ultrasonic sensor to measure water level and it is connected to a data acquisition board and this, in turn, is connected to a wireless system. The wireless system is an MDA300CA unit manufactured by Crossbow Technology (Milpitas, CA, USA) and uses IEEE 802.15 standard to send the information to the data processing module.
- The *Network Video Recording Module* is composed of a group of cameras installed at main intersections. Cameras provide traffic monitoring information in video and images. This system includes four Redeye Z205 network cameras and can be connected via Ethernet to the data processing module. Each camera has an IP address assigned to which users will have access from any Web searcher.
- The *Data Processing Module* combines all sources of information. This module provides three types of information: raw data, predicted data, and video information. The raw data is the information obtained by the sensors, while the predicted data are obtained through mathematical models. All of this must be accessible online.

### 4.2. Barranquilla, Colombia

**Figure 3:**
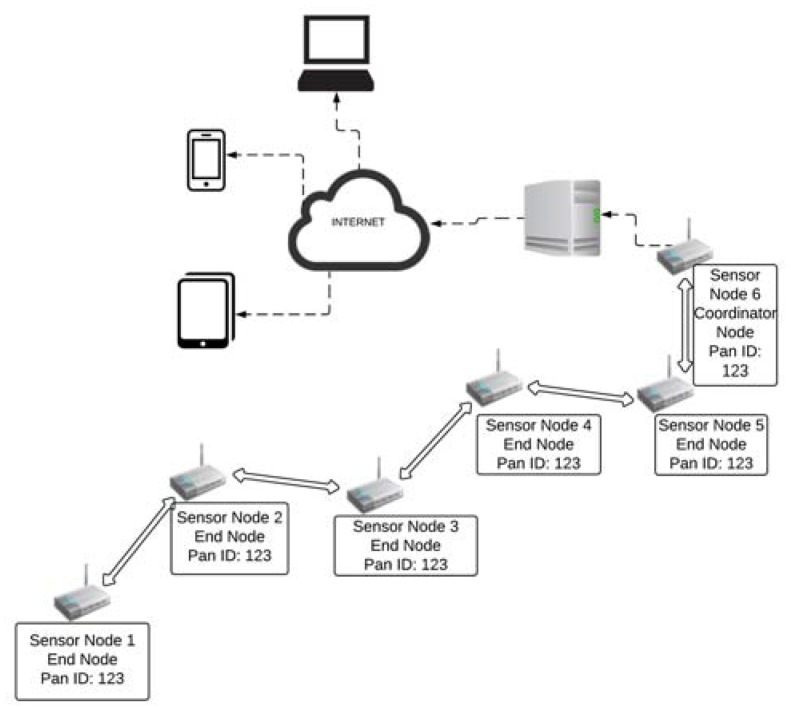
Wireless Sensor Network (WSN) architecture in the Barranquilla (Colombia) project.

The city of Barranquilla is located in the Caribbean Region of Colombia and does not have an efficient rainwater drainage system; therefore, during rainy events, streets become dangerous streams called “arroyos” [70,71]. Researchers at the Universidad de la Costa developed an EWS with a wireless sensor network and a WEB application [72]. Figure 3 illustrates the Wireless Sensor Network (WSN) architecture in the Barranquilla (Colombia) project.

- The *wireless sensor network* has six nodes and each node has a temperature, humidity and atmospheric pressure sensor connected to a mote (Waspmote from Libelium, Zaragoza, Spain) and powered by a photovoltaic system. This system was used by Ramírez-Cerpa et al. [73] to determine through an analysis the influence of the variation of these atmospheric variables in the formation of precipitations that cause flash floods in the city of Barranquilla. Information obtained via nodes is sent to a server using Zigbee technology with the XBee-PRO ZB (S2) radio module [74]. This module uses ZigBee technology under the IEEE 802.15.4 standard to communicate with other nodes and with the base station. Previously, in Caicedo-Ortíz et al. [75], a test was conducted to verify the transmission range of the Waspmote pro. It established an efficient communication between the transmitter node and the receiving node at a distance of 1000 m with line of sight.
- A server receives the data from the wireless sensor network and, through a Web and mobile application, gives information to end-users.

### 4.3. Manila, Philippines

In two streets near the Manila subway, a real-time urban flood monitoring system was installed [76]. A flood prediction model was developed to identify flooded streets and alternative routes for drivers. The system is divided into three main sections: Electronic instrumentation, Server and Web services.

**Figure 4:**
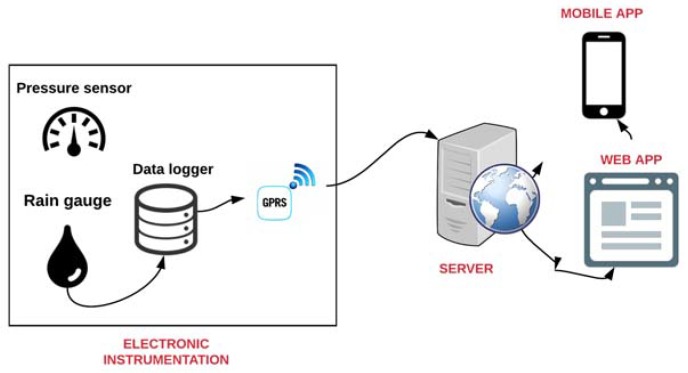
Urban Flood Monitoring System for Manila (Philippines) Metro project.

- The *Electronic Instrumentation* has a ground-based pressure sensor and a tipping bucket rain gauge connected to the data logger and powered by a photovoltaic system. The obtained information is sent through a General Packet Radio Service (GPRS) module to a server. Two nodes were installed on two nearby streets (Earnshaw and San Diego) on Boulevard Spain, Manila.
- The *Server* receives the data and processes it to provide real-time information. A Web application provides real-time information, historical data and flood data to users. Likewise, a mobile application shows the real-time variation of flash floods in the streets so that users can adjust their routes and travel schedules. Figure 4 illustrates the urban flood monitoring system for Manila (Philippines) Metro project.

### 4.4. Nakhon Si Thammarat, Thailand

**Figure 5:**
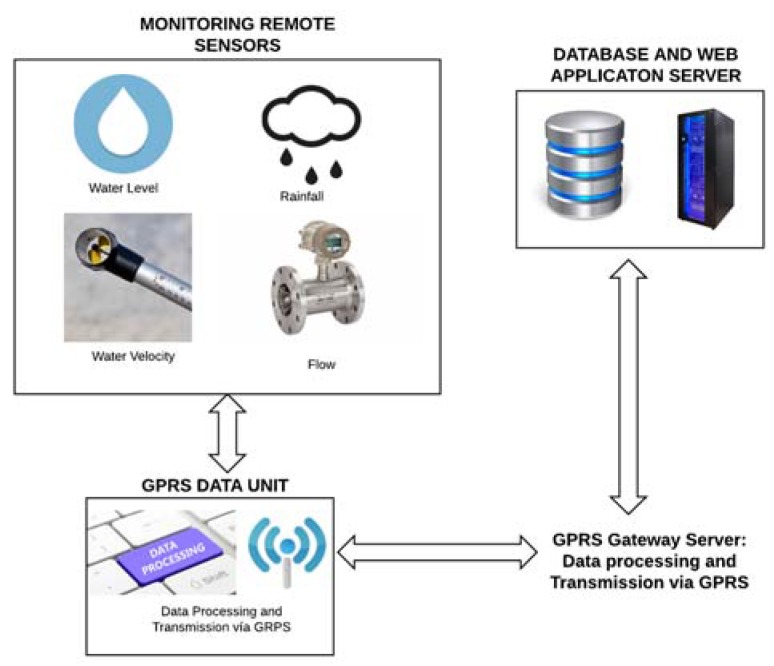
Wireless flood monitoring system implemented in the Nakhon Si Thammarat project [77].

In Nakhon Si Thammarat, a province in southern Thailand, a wireless flood monitoring system was developed for the mitigation and management of flood disasters in urban and suburban areas [77]. The system consists of two main modules, Remote Site and Control centre, as shown in Figure 5.

- The *Remote Site*. The monitoring section contains 15 remote devices located around the Nakhon Si Thammarat flood risk zone. A tipping bucket rain gauge was used to measure the amount and intensity of the rain. These remote devices use an ultrasonic Doppler instrument called STARFLOW (Unidata, Perth, Australia) to measure water level and velocity. Since the STARFLOW equipment is very sensitive to fluctuations in water velocity in the channel, the average velocity was used in a time interval rather than raw measurement data. The STARFLOW unit is connected to the GPRS Data Unit (GDU) and sends the information every 10 min to the control centre
- The *Control Center* has a server that contains the historical database, processes in real time the information and displays it through a WEB application. End-users can access this system through the Internet or mobile devices. The alert messages are also sent via text messages (SMS), FAX and email to the community.

To avoid unexpected power disruptions, an uninterruptible power supply (UPS) and a surge protector was installed. This allows the whole equipment to work for at least 24 h with a continuous electrical energy supply when not available.

### 4.5. Mayagüez, Puerto Rico

The University of Puerto Rico, Mayagüez (UPRM) campus developed a weather radar network that provides accurate and real-time hydro-meteorological information to the west region of the island [78,79]. These radars have a temporal resolution of 3 min, spatial resolution of 15 m and operate at a frequency of 9.1 GHz. The information obtained by the radars is sent to a data centre placed at Mayagüez campus with a high-performance directional grid parabolic antenna with a frequency of 2.4 GHz. A photovoltaic system provides the power supply to the radars and the data is deployed in a Web application. 

This weather radar network information was used to develop a flood alert system in western Puerto Rico for convective precipitation of time periods of a few hours or less (nowcasting) [80]. 

Weather radars provide information on cloud reflectivity and this data can be transformed into rainfall amount using empirical equations. There is an empirical relationship between the amount of precipitation and radar reflectivity, which in turn depends on the distribution of raindrops. The Rain Rate R (mm/h) is related with the reflectivity factor Z (mm^6^ m^−3^) through the Marshall–Palmer [81] equation:

(1) $$R\;\left(\frac{\mathrm{mm}}{h}\right)=0.036\times 10^{0.625*\mathrm{dBZ}}\;$$

Knowing the precipitation rate of different hydrological models for the prevention of floods can be developed thus enabling the community to be informed opportunely. Likewise, the aeronautical operations can be planned with greater precision [71].

Torres-Molina [80] used equation 1 to obtain the precipitation rate from weather radars and routed through a rainfall-runoff model *Vflo.* Using a coupled rainfall-runoff forecasting procedure obtained results with lead-times of 10, 20 and 30 min. These results were analyzed and compared using statistical methods. The flooding model Inundation Animator showed the extent of flooding superimposed onto a land map.

### 4.6. Barcelona, Spain

Llort et al. [82] presented a pluvial flood EWS, called FloodAlert, based on the use of radar observations to issue local flood warnings. This project, like the one developed in Mayaguez (Puerto Rico), uses the radar data and through the climatological Z–R relationship converts the reflectivity measurement into the amount of precipitation (mm/h).

Due to different errors affecting radar precipitation data, this project implemented a quality control process that includes statistical calibration of radar reflectivity estimations, correction of non-meteorological echoes and correction for underestimation due to beam blockages.

This project not only provides real-time radar information but also the precipitation movement field can be calculated using the last radar observations by means of cross-correlation techniques. Once both the radar data and radar nowcasting is available, the system calculates the 30 min accumulation in a moving window scheme.

To visualize the information, a web platform dynamically displays geo-referenced information of real-time radar observations and nowcasting. Likewise, the areas are shown that will be potentially affected by rainfall accumulation in 30 min exceeding the user-defined thresholds and the evolution of the maximums of the 30 min accumulation in the intelligent area surrounding the point of interest.

In order to send the alerts, this system uses email and text messages and the devices can be configured under different profiles (e.g., standard, 24 h, weekends, emergency, etc.). For example, on 29 October 2013 for an observation point in Palma de Mallorca, the forecasting accumulation values (30 min accumulation) exceeded the user-defined red threshold and an email was sent 90 min before the flooding at the city caused several problems. In the email, the top panel shows the areas forecasted to be over the thresholds (5, 10 and 20 mm/30 min in this case) and how those areas affect the point centred in the city. 

Unlike the other projects reviewed, this one does not describe the type of communication used to send the information to the data processing centre, or if the data was processed at the radar installation site. Similarly, it does not describe the power supply system.

Table 4 summarizes the instruments implemented in the projects mentioned above to measure the variables related to the formation of pluvial flash floods. Likewise, Table 4 shows the communication protocols to send sensors’ data, the alert dissemination methods and the power supply system.

All the reviewed projects focused their early warning system design on the forecasting and alert dissemination processes. The project developed in Nakhon Si Thammarat (Thailand) included the greatest number of hydrological and hydraulic variables for establishing alert levels. Likewise, this project used more than three communication channels to send alerts but it was the only one that was connected to the electrical grid.

## 5. Discussion

With regard to pluvial flash floods, for an early warning system to be effective, the alerts must be issued timely, be clear and understandable to the entire community at risk. Through this review, the key elements of an EWS for flash floods in urban areas were described as well as the variables that influence their formation.

Early Warning Systems implemented in different locations worldwide were reviewed to identify the main elements used for the forecasting process such as measurement instruments, data transmission protocols and power supply equipment, as well as information processing methods. Also reviewed were the means and strategies for alert dissemination to the community. 

### 5.1. Forecasting Process

The forecasting process is linked to the detection, monitoring and analysis of meteorological and hydraulic variables related to flash floods. It can be carried out using various instruments and methods, but there are indispensable devices for this work. 

From the reviewed projects, three considered the amount of rain (mm) as a basic element for the development of alerts for urban flash floods. The most used instrument for direct precipitation measurement is the rain gauge and for indirect measurement is weather radars. 

Rain gauges measure the liquid precipitation expressed in mm during a period of time. Once this measure is registered, it is sent to an information-processing centre. The reviewed projects implemented tipping bucket rain gauges and consist of a light metal container or bucket divided into two compartments. The liquid precipitation is collected into the uppermost compartment and, after a predetermined amount has entered, the bucket becomes unstable and tips toward its alternative rest position [83].

Weather radars are widely used instruments to locate precipitation, identify the types and monitor their movements. This instrument emits microwave pulses and measures the reflected signal from the raindrops [84]; the higher the reflected signal value, the higher the rain intensity.

One of the advantages of weather radars is that they have a higher coverage than rain gauges. Since they can also monitor the movement of the clouds, weather radars can predict phenomena ahead of time, and serve as a backup system in case the on-site devices are removed by flooding or high winds. However, weather radars are more expensive than rain gauges, have higher power consumption and need more technical and social requirements for their implementation.

Three projects included sensors for measuring the water level and can be classified into pressure sensors and ultrasonic sensors. Pressure sensors measure the uniform weight of a column of water. Since weight is a force, a column of water with a specific height will always exert the same amount of pressure on the sensor. At the output, the sensor produces a voltage equivalent to the received pressure and then this voltage value translates it to a level measurement [85].

Ultrasonic sensors send a sound wave with a specific frequency to an object and receive the reflected sound wave. The sensor measures the distance by calculating the sending and receiving time of this sound wave [86]. Ultrasonic sensors are not affected by colour, transparency of objects, design or type of surface. They are resistant to external disturbances such as vibration and ambient noise. These sensors have great accuracy and they are easy to connect with different interfaces [87]. However, environmental variables such as air temperature and humidity can affect the echo transit time and therefore the measurement accuracy of an ultrasonic sensor [88].

Ultrasonic wave propagation speed depends on both the nature of the propagation medium and the temperature. When the air temperature and humidity increase, the speed of sound increases and the reach is shorter. This reduction is not linear and differs from sensor to sensor [89].

For some applications, one of the disadvantages of the ultrasonic sensor is that it cannot work underwater, but for flooding applications, this is suitable because the streams sweep away different objects that can collide with the sensor, introducing wrong measurements and causing damage to the equipment. Thus, it is suggested that non-submersible sensors be used for the implementation of early warning systems for flash floods in urban areas.

For the communication technology implemented to send the sensors’ data, most of the reviewed projects used wireless communication. Wireless modules under IEEE 802.15 standard and GPRS modules were implemented to send the information from the measurement stations to a data centre. However, these projects only implemented one communication channel for sending the data. If there are failures in the communication system, the alert will not be timely. Therefore, it is necessary to have a minimum of two communication channels for sending the alerts.

Ch. Saad et al. [90] performed a comparative analysis of wireless communication protocols for intelligent sensors with a focus on their performance. Table 5 presents the differences between some wireless communication protocols in terms of the frequency band, the range of coverage, max data rate and transmitted power. 

The transmission time of a wireless system depends on the data rate, the message size, and the distance between two nodes [91]. From Table 5, GSM/GPRS has the lowest data rate, therefore, its transmission time is longer than the other protocols. Likewise, GSM/GPRS has the highest power transmission consumption, but it has the best range of coverage from these protocols. 

During high rainfall events, the power supply may fail. It is recommended to have a photovoltaic system connected to the equipment as a primary source of power supply or as a backup system in case of failure. Almost all projects use photovoltaic systems to supply electricity for measuring and communication instruments. Only one project was connected to the electric power grid and used a UPS as a protection measure.

Information processing is carried out in a data centre equipped with applications, and analysis software necessary for alert design. The data centre processes the sensor data and transforms it into alerts in real time. Some of these data centres have a historical database and provide online access to them like the projects implemented in Nakhon Si Thammarat (Thailand) and Florida (United States).

**Figure 6:**
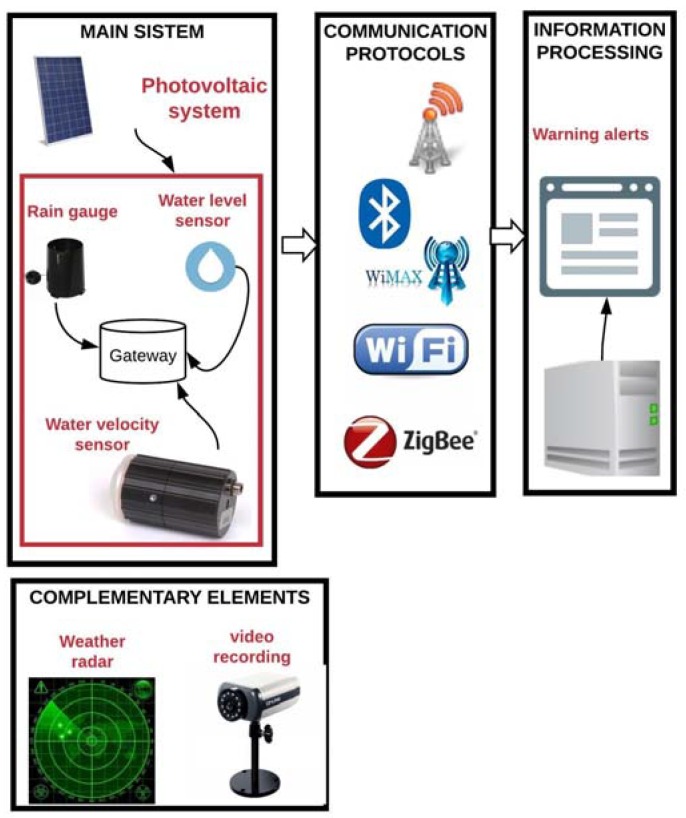
Consolidated forecasting process structure with main and complementary elements.

Figure 6 consolidates the overall structure of the forecasting process with the main and secondary elements. It also shows the communication protocols used in the reviewed projects to transmit the information from sensors to a data centre.

The measurement of water velocity as a hydraulic variable should be included in all pluvial flash flood EWS. The parameters, water level, water velocity and their combined effect, are responsible for the stability loss of pedestrians and drivers when trying to cross hazardous streams.

### 5.2. Dissemination Process

The dissemination of information must be timely, gathered and understood by the whole community. Warnings must be simple, clear and useful messages so that opportune decisions may be taken. It is necessary to have an integrated system that allows the information to be sent through different channels, ensuring that it is received and understood by everybody. 

Studies by Fakhruddin et al. [56] and Lam et al. [31] agree that the preferred channel for receiving information is television. However, younger people prefer to receive information through digital media and the use of social networks. Older people show their preference for using radio and audio alerts such as sirens.

The most used methods for sending alerts to the community at risk were Web and mobile applications. Four projects developed a Web application to visualize the alerts, water level and precipitation measurements. One of these projects developed a mobile application too. 

Another project offers online access to raw data and video information. However, none of the projects integrated television or radio to send the alerts.

According to WMO, the alerts should be brief, concise and understandable [5], but during high precipitation events it is also necessary to send the alerts through different communication channels. This will avoid the loss of messages due to channel failures; nevertheless, just two projects had more than one media for sending alerts.

**Figure 7:**
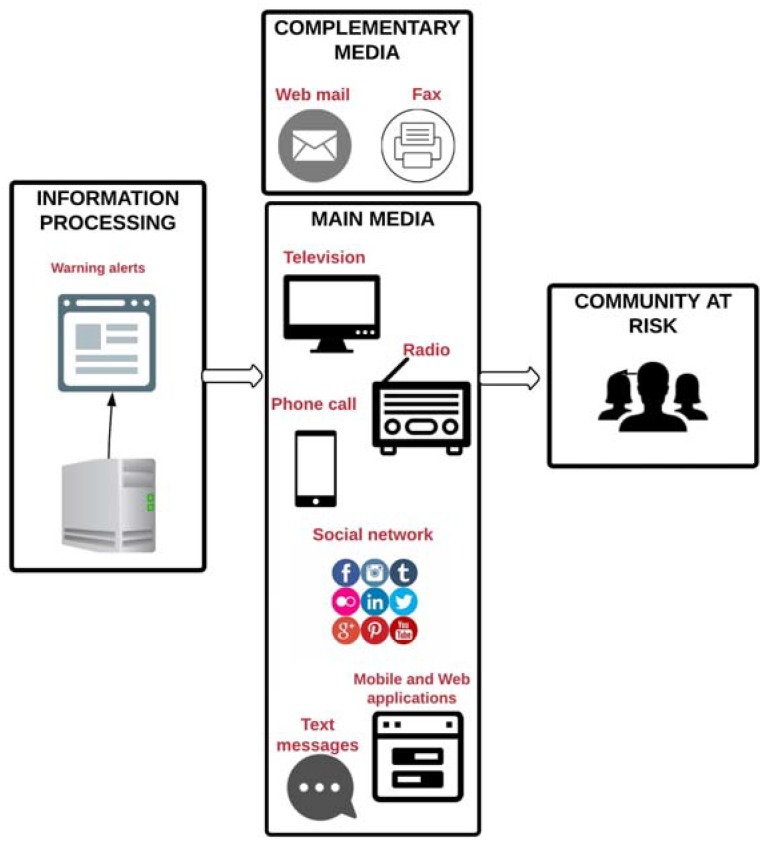
Main and complementary media used in reviewed projects for alert dissemination.

Television and radio were not included in any of the reviewed projects for alert dissemination, but they are very useful media for broadcasting messages to a large part of the population at risk. Likewise, an up-to-date system that visualizes streets during floods and applications that provide alternate routes for drivers is ideal. Figure 7 shows the different media that can be used for the dissemination of alerts according to this review.

**Figure 8:**
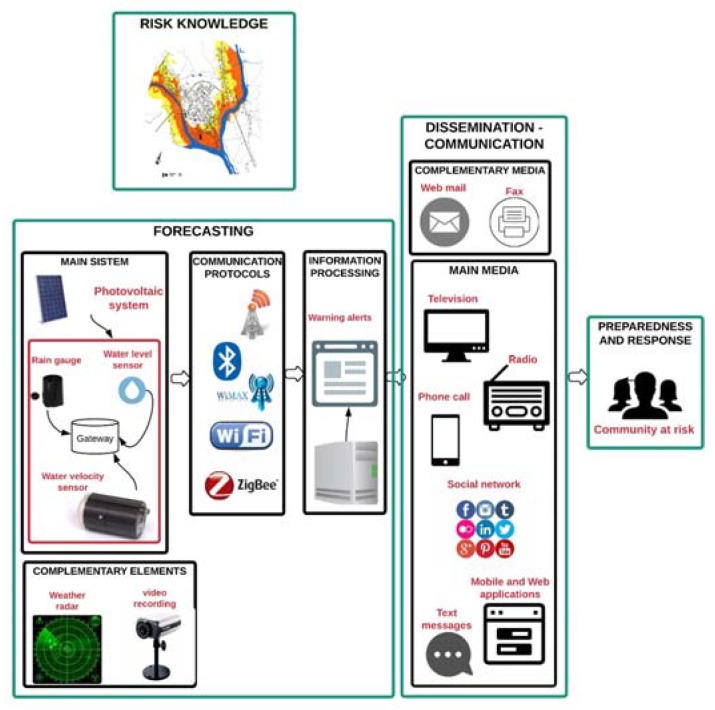
Key elements of the proposed pluvial flash flood early warning system.

Taking into account the guidelines provided in Section 3 for each key element of a pluvial flash flood EWS and the instruments, methods and media implemented in the reviewed projects for forecasting and alert dissemination, an effective and real-time pluvial flash flood Early Warning System is proposed. Figure 8 shows the main and secondary elements of each key element of the pluvial flash flood EWS proposed in this review.

Disaster risk knowledge is a necessary phase, prior to EWS design. It comprises identification and mapping of the risk. First, hazards, exposure, vulnerabilities and risk in the population are identified. This information must be obtained from official sources and must be relevant and concise. The next step is to develop a risk map to prioritize the EWS’s needs and guide preparations for disaster prevention and response. 

The forecasting process is divided into four sections: main section, complementary elements, communication protocols and information processing. The main section is compounded by water level and water velocity sensors and the rain gauge. This set is powered by a photovoltaic system. Radar and video cameras can be considered as complementary elements. Weather radars are the most suitable instruments for monitoring during extreme rainfall events but they are quite expensive. Figure 8 presents different wireless communication protocols to send the information to the main system. It is necessary to implement at least two different protocols for redundancy. In case of failures in one protocol, the data can be sent timely to the information-processing centre and without loss of packages. This centre is in charge of the data processing and designs the flood warnings.

Once the alerts are ready, there are different media to send them. Web and mobile applications were implemented in all the reviewed projects to visualize the alerts, but it is necessary to have more than one channel to cover the entire community at risk. Television is the preferred media to receive the alerts, but government support is needed to be able to send broadcast messages [56].

Sending warning messages to the community at risk is not the last action in an EWS. The communication is established when people receive, understand the message and take timely decisions. To achieve this, it is necessary that the community and the local authorities participate actively in the decision-making process. One proposal on this topic is the project named FloodCitiSense “Early warning service for urban pluvial floods for and by citizens and city authorities”. The aim of this project is to reduce urban areas and citizen vulnerability to pluvial floods. They propose integrating crowdsourced hydrological data measured by different participants such as citizens, local authorities, research units and industrial partners. Furthermore, they suggest implementing low-cost sensors and web-based technologies to display warnings [92]. This project will be developed during 2017–2020.

After implementation of an early warning system, it is very important to measure its performance in order to determine its effectiveness. Parker [93] mentioned the most common ways of measuring flood warning performance. They are classified in technical and socials measures. The following characteristics are related to technical measures:

- Probability of detection
- Accuracy: Forecast flood levels compared with measured flood levels.
- Reliability: Flood-hit, miss and false alarm rates.
- Probability (i.e., uncertainty): Amount or percentage of certainty/uncertainty associated with the forecast
- Time range ahead of the flood: How far ahead in time a forecast can be made
- Timeliness: Warning lead time
- Spatial resolution: The smallest area for which a forecast can be made

According to Parker [93], to measure the EWS acceptance by the community, social survey responses are required that consider the following characteristics:

- Warning information: Recipients’ assessments of the degree to which the warning provided them with the flood information they needed.
- Satisfaction with the flood warning service: Levels of satisfaction among those for whom flood warnings were/should have been provided.
- Damage Reduction: The amount of flood damage saved by the warning.
- Protection of life and limb: The assessed number of lives and injuries avoided by the warning.
- Benefit–cost ratio: The ratio of the assessed benefits and costs of providing a flood warning.

Some of the reviewed projects evaluated the EWS performance, considering only technical aspects. However, after their implementation, they did not register the level of acceptance by the community at risk or the damage reduction.

**Figure 9:**
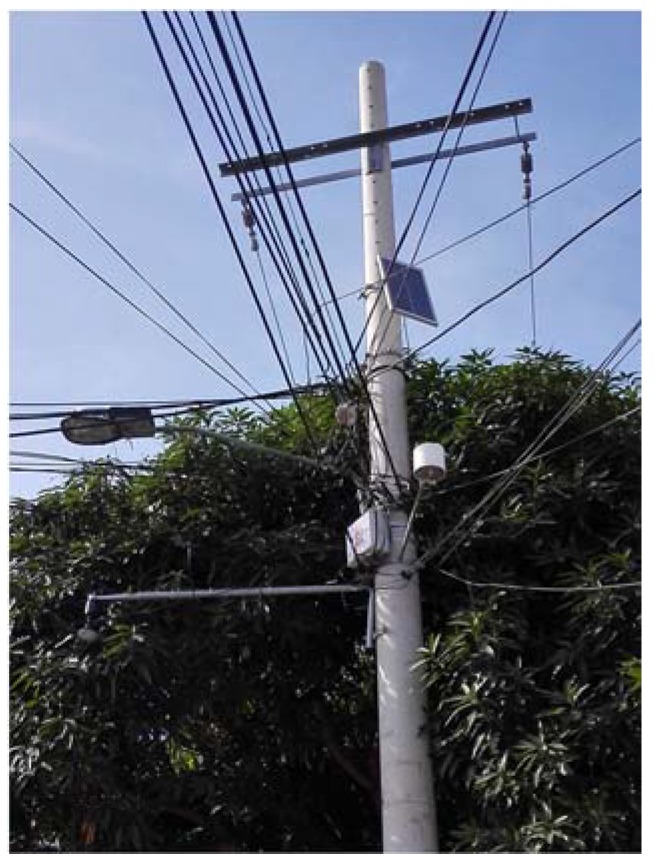
Monitoring, Communication and Power supply system of an EWS for urban flash floods in Barranquilla (Colombia)

The information obtained from this review study was applied to the development of an early warning system for detection in real-time of urban pluvial flooding hazard levels in an ungauged basin in Barranquilla, Colombia [23]. This design used the structure suggested in this study for the selection and installation of the main and complementary elements to measure in real-time the hydro-meteorological variables that influence the formation of urban flash floods. It also considered the types of sensors for measuring the water level and water velocity and the power supply system. Figure 9 illustrates the set of a water level sensor, a rain gauge, a gateway, and a photovoltaic and communication system for this project. 

This project developed a Web application considering the recommendations about the diffusion of information. The aim was to generate an effective and timely response from the population during flash floods. The information was updated every 5 min with the received precipitation value.

**Figure 10:**
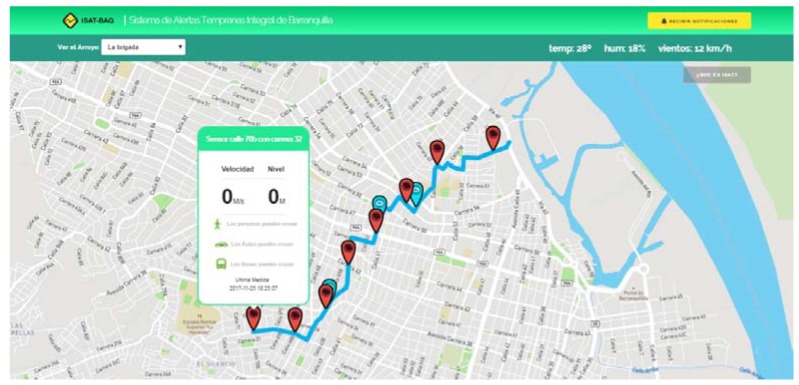
Web application (http: //www.isatbaq.com.co).

The application offers the option to subscribe to receive notifications during rainy events through the social networks Twitter and Telegram. Likewise, it allows the addition of more streams as well as the inclusion of as many observation points and rain gauges. In the future, it will display the atmospheric information obtained by different sensors. Figure 10 shows the Web application interface.

## 6. Conclusions

The effects of climate change have become evident in the increased formation of natural phenomena that can adversely affect people’s lives [94]. The increasing intensity and duration of rainfall in urban areas makes them more prone to flash floods, as the capacity of drainage systems is saturated, placing city inhabitants at risk and causing material losses. Flash floods, unlike other floods, are of very fast onset, with a relatively short spike and rapid withdrawal [94]. Therefore, it is necessary to design adequate and intelligent adaptation measures to reduce the negative impact on society.

EWS has been established worldwide as a useful tool for populations to adapt and mitigate the impact of flash floods in urban areas. Through this review, the basic architecture of EWS for flash floods in urban areas was determined. This EWS is people-centred and the community can have an active participation from design to implementation. The EWS is divided into four structures: Disaster Risk Knowledge, Forecasting, Dissemination and Communication of information and Preparedness and Response. 

Through this review, it was identified that the variables that must be monitored in real time during the rain events are the amount of rain and water level. The information of these variables is also processed in real time to issue alerts in a timely manner. Rain gauges, weather radars, ultrasonic and pressure sensors were the instruments implemented to measure these variables. Although weather radars have more coverage than rain gauges, they are more expensive and need more technical requirements for their implementation.

Since flash flood stream flow is turbulent and can wash away different objects in its path, the use of submersible sensors such as pressure sensors is not recommended. Therefore, ultrasonic or radar sensors are more suitable for flooring applications.

To send the sensor data measurements to a data centre, the reviewed projects used wireless communication systems; GPRS modules and wireless modules under 802.15 standard were the most used. GPRS modules have a better range of coverage than other wireless communication protocols; nevertheless, they have higher power consumption and longer transmission time.

This article has shown that not all the reviewed projects fully comply with the suggested norms for an effective early warning system. This article serves as a guide for the design of early warning systems for pluvial flash floods that affect urban areas, taking into account the instruments, protocols and primary and secondary means for the forecasting and alert dissemination process.

## Figures and Tables

**Table 1:** Preference of information sources during a disaster.

Source	Population Surveyed
Television	52%
Facebook	18.9%
WhatsApp	9.6%
Radio	8.2%
News Agency Websites	6.1%
Government Websites	2.9%

**Table 2:** Summary of respondents’ descriptions of actions that a person should take in response to a flash flood warning [67].

Action	% of Respondents	Example Public Response (s)
Move to a higher location	84%	“Climb to safety”
		“Run to higher ground”
		“Get to higher ground and hold on”
		“Climb a tree...”
		“Get to a multilevel building and get to the top”
		“Drive uphill, get out of the car and continue uphill on foot”
		“Get as high as possible”
Move to a different location	18%	“Drive to flatland, away from Boulder Creek away from mountains and to higher land”
		“Run like nuts”
		“Get to nearest safety shelter, hospital, firehouse”
Avoid risky areas	12%	“Stay away from creeks + rivers”
		“Move away from creek areas”
		“Find higher ground away from electric lines”
Go inside	10%	“Get inside a strong building”
		“Go in a commercial building or knock on a door”
Assess situation	4%	“Think! Assess the vulnerability of location and act accordingly...”
		“Determine if the flood will be in your area and take appropriate action”
		“Have high ground picked out nearby and go to it if you see the water and debris coming”
Be alert	3%	“Raise alert level and make a plan for possible action”
		“Be aware of nearby floodways/drainages”
Seek more information	1%	“Try to obtain more info about where to go for safety
Depends	7%	“Go to a higher place or leave the area if there is time”
		“It depends on where you are?”
Don’t know	1%	“Honestly, I have no idea”
Other	8%	“Check to hear if it is a practice warning or a real one—then call loved ones and go to a safe location”
		“Call for help and look for high ground”

**Table 3:** Key elements and Key actors of an Early Warning System [55].

Key Element	Key Actors
Disaster risk knowledge	1. International, national and local disaster management agencies.
	2. Meteorological and hydrological organizations.
	3. Geophysical experts
	4. Social scientists
	5. Engineers
	6. Land use and urban planners
	7. Researchers and academics
	8. Organizations and community representatives involved in disaster management
Forecasting	1. National meteorological and hydrological services
	2. Specialized observatory and warning centres
	3. Universities and research institutes
	4. Private sector equipment supplier telecommunications authorities
	5. Quality management experts
	6. Regional technical centres
Dissemination and communication	1. International, national and local disaster management agencies
	2. National meteorological and hydrological services
	3. Military and civil authorities
	4. Media organizations (print, television, radio and online)
	4. Businesses in vulnerable sectors (e.g., tourism, aged care facilities, marine vessels)
	5. Community-based and grassroots organizations
	6. International and local agencies
Preparedness and response	1. Community-based and grassroots organizations
	2. Schools, universities and informal education sector.
	3. Media (print, radio, television, online)
	4. Technical agencies with specialized knowledge of hazards
	5. International, national and local disaster management agencies

**Table 4:** Instruments, communication protocols and methods for alert dissemination.

Location	Sensors		Communication System	Alert Dissemination	Power Supply
	Type	Variables to Measure			
Nakhon Si Thammarat, Thailand	STARLFLOW Ultrasonic Doppler sensor	Water level and velocity	GPRS module	Web application. SMS, FAX, email.	Connected to the electrical grid and UPS
	Tipping bucket rain gauge	Amount of rain			
Florida, United States	Ultrasonic sensor WL700	Water level	Wireless unit (IEEE 802.15)	Online access to raw and predicted data, video information	Photovoltaic system
	Redeye Z205 Cameras		Ethernet		
Barranquilla, Colombia	Humidity sensor	Atmospheric variables	ZigBee (IEEE 802.15)	Web and mobile application	Photovoltaic system
	Temperature sensor				
	Atmospheric pressure				
Manila, Philippines	Pressure sensor	Water level	GPRS module	Web application	Photovoltaic system
	Tipping bucket rain gauge	Amount of rain			
Mayagüez, Puerto Rico	Weather radar	Radar reflectivity and amount of rain	Parabolic antenna (IEEE 802.15)	Web application	Photovoltaic system
Barcelona, Spain	Weather radar	Radar reflectivity and amount of rain		Web application, SMS, E-mail	

**Table 5:** Differences between wireless communication protocols.

Protocols	Bluetooth	Ultrawide Band (UWB)	ZigBee/IP	Wi-Fi	Wi-Max	GSM/GPRS
**Frequency band**	2.4 GHz	3.1–10.6 GHz	868/915 MHz; 2.4 GHz	2.4; 5 GHz	2.4; 5.1–66 GHz	850/900; 1800/1900 MHz
**Nominal range**	10 m	10–102 m	10–1000 m	10–100 m	0.3–49 km	2–35 km
**Max data rate (Mbit/s)**	0.72	110	0.25	54	70	0.168
**Bit time (μs)**	1.39	0.009	4	0.0185	0.0143	5.95
**Transmitted Power (W)**	0.1	0.04	0.0063	1	0.25	2

## References

[1] Kundzewicz Z.W. (2002). Non-structural flood protection and sustainability. Water Int..

[2] Singh P., Sinha V.S.P., Vijhani A., Pahuja N. (2018). Vulnerability assessment of urban road network from urban flood. Int. J. Disaster Risk Reduct..

[3] Birkmann J., von Teichman K. (2010). Integrating disaster risk reduction and climate change adaptation: Key challenges—Scales, knowledge, and norms. Sustain. Sci..

[4] International Strategy for Disaster Reduction (ISDR) Emerging Challenges for Early Warning Systems in context of Climate Change and Urbanization. http://www.preventionweb.net/files/15689_ewsincontextofccandurbanization.pdf.

[5] Chaumillon E., Bertin X., Fortunato A.B., Bajo M., Schneider J.-C., Dezileau L., Walsh J.P., Michelot A., Chauveau E., Créach A. (2017). Storm-induced marine flooding: Lessons from a multidisciplinary approach. Earth Sci. Rev..

[6] The University Corporation for Atmospheric Research (2010). Flash Flood Early Warning System Reference Guide.

[7] National Weather Service (2017). Flood Safety Awareness Week: Flood Hazards. http://www.weather.gov/aly/fldsafetyWednesday.

[8] Ministère de l’Écologie and du Développement Durable et de l’Énergie (2016). Submersion Marine.

[9] Jha A.K., Bloch R., Lamond J., The World Bank (2012). Cities and Flooding A Guide to Integrated Urban Flood Risk Management for the 21st Century.

[10] Alfieri L., Cohen S., Galantowicz J., Schumann G.J.-P., Trigg M.A., Zsoter E., Prudhomme C., Kruczkiewicz A., de Perez E.C., Flamig Z. (2018). A global network for operational flood risk reduction. Environ. Sci. Policy.

[11] Maggioni V., Massari C. (2018). On the performance of satellite precipitation products in riverine flood modeling: A review. J. Hydrol..

[12] Da Cruz Simoes N.E. (2012). Urban Pluvial Flood Forecasting.

[13] Jiang Y., Zevenbergen C., Ma Y. (2018). Urban pluvial flooding and stormwater management: A contemporary review of China’s challenges and ‘sponge cities’ strategy. Environ. Sci. Policy.

[14] Ten Veldhuis J.A.E. (2011). How the choice of flood damage metrics influences urban flood risk assessment. J. Flood Risk Manag..

[15] World Meteorological Organization (2017). Global Approach to Address Flash Floods. http://www.hrc-lab.org/publicbenefit/downloads/wmo-flashflood.pdf.

[16] World Meteorological Organization (2010). Guidelines on Early Warning Systems and Application of Nowcasting and Warning Operations.

[17] Chen Y., Zhou H., Zhang H., Du G., Zhou J. (2015). Urban flood risk warning under rapid urbanization. Environ. Res..

[18] Intergovernmental Panel on Climate Change (2014). Climate Change 2014—Impacts, Adaptation and Vulnerability: Part B: Regional Aspects: Working Group II Contribution to the IPCC Fifth Assessment Report.

[19] European Commission and Water Group Floods (WGF) (2016). Pluvial Flooding: An EU Overview.

[20] Guerreiro S.B., Glenis V., Dawson R.J., Kilsby C. (2017). Pluvial Flooding in European Cities—A Continental Approach to Urban Flood Modelling. Water.

[21] Houston D., Werritty A., Bassett D., Geddes A., Hoolachan A., McMillan M. (2011). Pluvial (Rain-Related) Flooding in Urban Areas: The Invisible Hazard.

[22] Bhattarai R., Yoshimura K., Seto S., Nakamura S., Oki T. (2016). Statistical model for economic damage from pluvial floods in Japan using rainfall data and socioeconomic parameters. Nat. Hazards Earth Syst. Sci..

[23] Acosta-Coll M., Ballester-Merelo F., Martinez-Peiró M. (2018). Early warning system for detection of urban pluvial flooding hazard levels in an ungauged basin. Nat. Hazards.

[24] Zhang W., Li S.M., Shi Z. (2012). Formation causes and coping strategies of urban rainstorm waterlogging in China. J. Nat. Disasters.

[25] Yin J., Ye M., Yin Z., Xu S. (2015). A review of advances in urban flood risk analysis over China. Stoch. Environ. Res. Risk Assess..

[26] Azam M., Kin H.S., Maeng S.J. (2017). Development of flood alert application in Mushim stream watershed Korea. Int. J. Disaster Risk Reduct..

[27] Creutin J.D., Borga M., Gruntfest E., Lutoff C., Zocatelli D., Ruin I. (2013). A space and time framework for analyzing human anticipation of flash floods. J. Hydrol..

[28] Yin J., Yu D., Yin Z., Liu M., He Q. (2016). Evaluating the impact and risk of pluvial flash flood on intra-urban road network: A case study in the city center of Shanghai, China. J. Hydrol..

[29] International Strategy for Disaster Reduction (ISDR) UNISDR Terminology on Disaster Risk Reduction. https://www.unisdr.org/we/inform/publications/657.

[30] Einfalt T., Hatzfeld F., Wagner A., Seltmann J., Castro D., Frerichs S. (2009). URBAS: Forecasting and management of flash floods in urban areas. Urban Water J..

[31] Lam R., Leung L.P., Balsari S., Hsiao K.-H., Newnham E., Patrick K., Pham P., Leaning J. (2017). Urban disaster preparedness of Hong Kong residents: A territory-wide survey. Int. J. Disaster Risk Reduct..

[32] Grasso V., Singh A., Pathak J., United Nations Environment Programme (2012). Early Warning Systems a State of the Art Analysis and Future Directions.

[33] Bouwer L., Papyrakis E., Poussin J., Pfurtscheller C., Thieken A. (2014). The costing of measures for natural hazard mitigation in Europe. Nat. Hazards Rev..

[34] Praskievicz S., Chang H. (2009). A review of hydrological modelling of basin-scale climate change and urban development impacts. Prog. Phys. Geogr..

[35] Hunt A., Watkiss P. (2011). Climate change impacts and adaptation in cities: A review of the literature. Clim. Chang..

[36] Kundzewicz Z.W., Kanae S., Seneviratne S.I. (2013). Flood risk and climate change: Global and regional perspectives. Hydrol. Sci. J..

[37] You Q., Kang S., Aguilar E. (2011). Changes in daily climate extremes in China and their connection to the large scale atmospheric circulation during 1961–2003. Clim. Dyn..

[38] Ding Y.H., Ren G.Y., Shi G.Y. (2016). National assessment report of climate change (I): Climate change in China and its future trend. Adv. Clim. Chang. Res..

[39] Liu Z., Xia J. (2016). Impact of climate change on flood disaster risk in China. Chin. J. Nat..

[40] Office for National Statistics (ONS) (2014). Population Projections: 2014-Based Statistical Bulletin. https://www.ons.gov.uk/peoplepopulationandcommunity/populationandmigration/populationprojections/bulletins/nationalpopulationprojections/2015-10-29).

[41] National Bureau of Statistics of China (NBSC) (2016). China Statistical Yearbook 2016.

[42] Miller J.D., Hutchins M. (2017). The impacts of urbanisation and climate change on urban flooding and urban water quality: A review of the evidence concerning the United Kingdom. J. Hydrol. Reg. Stud..

[43] Borga M., Anagnostou E.N., Blöschl G., Creutin J.D. (2011). Flash flood forecasting, warning and risk management: The HYDRATE project. Environ. Sci. Policy.

[44] Grillakis M.G., Koutroulis A.G., Komma J., Tsanis I.K., Wagner W., Blöschl G. (2016). Initial soil moisture effects on flash flood generation—A comparison between basins of contrasting hydro-climatic conditions. J. Hydrol..

[45] Zhang J., Yu Z., Yu T., Si J., Feng Q., Cao S. (2018). Transforming flash floods into resources in arid China. Land Use Policy.

[46] United Nations Office for Disaster Risk Reduction (UNISDR) (2004). Living with Risk a Global Review of Disaster Reduction Initiatives.

[47] Spiekermann R., Kienberger S., Norton J., Briones F., Weichselgartner J. (2015). The Disaster-Knowledge Matrix—Reframing and evaluating the knowledge challenges in disaster risk reduction. Int. J. Disaster Risk Reduct..

[48] Weichselgartner J., Pigeon P. (2015). The Role of Knowledge in Disaster Risk Reduction. Int. J. Disaster Risk Sci..

[49] Hunt D.P. (2003). The concept of knowledge and how to measure it. J. Intellect. Cap..

[50] United Nations Development Programme (2000). Energy and the Challenge of Sustainability.

[51] The Intergovernmental Panel on Climate Change (IPCC) (2012). Managing the Risks of Extreme Events and Disasters to Advance Climate Change Adaptation. Special Report of the Intergobernmental Panel on Cimate Change.

[52] United Nations Development Programme (UNDP) and Regional Crisis Prevention and Recovery Programme (2008). Strengthening Capacities for Disaster Risk Reduction, A Primer. https://www.preventionweb.net/files/globalplatform/entry_bg_paper~strengtheningcapacityfordrraprimerfullreport.pdf.

[53] Unidad Nacional Para la Gestión del Riesgo de Desastres (UNGRD) and Programa de las Naciones Unidas Para el Desarrollo (PNUD) (2012). Guía Metodológica Para la Elaboración de Planes Departamentales Para la Gestión del Riesgo. http://repositorio.gestiondelriesgo.gov.co/handle/20.500.11762/20871?show=full.

[54] Surjan A., Sharma A., Shaw R., Shaw A.S.R. (2011). Understandig Urban resilience. Community, Environment and Disaster Risk Management.

[55] International Strategy for Disaster Reduction (ISDR) and German Committee for Disaster Reduction Developing Early Warning Systems: A Checklist. Proceedings of the Third International Conference on Early Warning (EWC III).

[56] Fakhruddin S.H.M., Kawasaki A., Babel M.S. (2015). Community responses to flood early warning system: Case study in Kaijuri Union, Bangladesh. Int. J. Disaster Risk Reduct..

[57] Balis B., Kasztelnik M., Bubak M., Bartynski T., Gubał T., Nowakowski P., Broekhuijsen J. (2011). The UrbanFlood common information space for early warning systems. Procedia Comput. Sci..

[58] Krzhizhanovskaya V.V., Shirshov G.S., Melnikova N.B., Belleman R.G., Rusadi F.I., Broekhuijsen B.J., Gouldby B.P., Lhomme J., Balis B., Bubak M. (2011). Flood early warning system: Design, implementation and computational modules. Procedia Comput. Sci..

[59] Chang C.L.-H., Lin T.-C. (2015). The role of organizational culture in the knowledge management process. J. Knowl. Manag..

[60] Mark O., Weesakul S., Apirumanekul C., Boonya-Aroonet S., Djordjević S. (2004). Potential and limitations of 1D modelling of urban flooding. J. Hydrol..

[61] Henonin J., Russo B., Mark O., Gourbesville P. (2013). Real-time urban flood forecasting and modelling—A state of the art. J. Hydroinform..

[62] Mayhorn C., Collins A. (2014). Warning the world of extreme events: A global perspective on risk communication for natural and technological disaster. Saf. Sci..

[63] Cools J., Innocenti D., O’Brien S. (2016). Lessons from flood early warning systems. Environ. Sci. Policy.

[64] Plate E.J. (2007). Early warning and flood forecasting for large rivers with the lower Mekong as example. J. Hydro-Environ. Res..

[65] Nezih A., Green W. (2006). OR/MS research in disaster operations management. Eur. J. Oper. Res..

[66] Alfieri L., Burek P., Dutra E., Krzeminksi B., Muraro D., Thielen J., Pappenberger F. (2013). GloFAS—global ensemble streamflow forecasting and flood early warning. Earth Syst. Sci..

[67] Morss R.E., Mulder K.J., Lazo J.K., Demuth J.L. (2016). How do people perceive, understand, and anticipate responding to flash flood risks and warnings? Results from a public survey in Boulder, Colorado, USA. J. Hydrol..

[68] Quevauiller P., Innocenti D. (2014). When Science Meets Policy: Enhancing Governance and Management of Disaster Risks. Hydrometeorological Hazards: Interfacing Science and Policy.

[69] Chang N., Guo D.-H. Urban flash flood monitoring, mapping and forecasting via a tailored sensor network system. Proceedings of the 2006 IEEE International Conference Networking, Sensing Control.

[70] UNGRD (2012). Guía Metodológica Para la Elaboración de Planes de Departamentales Para la Gestión del Riesgo. http://repositorio.gestiondelriesgo.gov.co/handle/20.500.11762/20871.

[71] Acosta-Coll M. (2013). Sistemas de Alerta Temprana (S.A.T) para la Reducción del Riesgo de Inundaciones Súbitas y Fenómenos Atmosféricos en el Área Metropolitana de Barranquilla. Sci. Tech..

[72] Cama-Pinto A., Piñeres-Espitia G., Zamora-Musa R., Acosta-Coll M., Caicedo-Ortiz J., Sepúlveda-Ojeda J. (2016). Design of a wireless sensor network for monitoring of flash floods in the city of Barranquilla, Colombia. Rev. Chil. Ingeniare.

[73] Ramírez-Cerpa E., Acosta-Coll M., Vélez-Zapata J. (2017). Analysis of the climatic conditions for short-term precipitation in urban areas: A case study Barranquilla, Colombia. Idesia.

[74] Piñeres-Espitia G., Mejía-Neira A. (2013). Plataformas tecnológicas aplicadas al monitoreo climático. Prospectiva.

[75] Caicedo-Ortiz J.G., Acosta-Coll M.A., Cama-Pinto A. (2015). Modelo de despliegue de una WSN para la medición de las variables climáticas que causan fuertes precipitaciones. Prospectiva.

[76] Garcia F.C.C., Retamar A.E., Javier J.C. A real time urban flood monitoring system for metro Manila. Proceedings of the IEEE Region 10 Conference Annual International Conference Proceedings/TENCON.

[77] Sunkpho J., Ootamakorn C. (2011). Real-time flood monitoring and warning system. Songklanakarin J. Sci. Technol..

[78] Acosta-Coll M. (2013). Radares Meteorológicos de Bajo Costo para la Detección de Precipitación y Desarrollo de Operaciones Aéreas en Colombia. Rev. Colomb. Tecnol. Av..

[79] Acosta-Coll M. (2011). Métodos de Eliminación de ecos Fijos y la Integración de los datos de una red de Radares Meteorológicos Banda-X en Terrenos Complejos.

[80] Torres-Molina L. (2014). Flood Alert System Using Rainfall Data in the Mayagüez Bay Drainage Basin, Western Puerto Rico.

[81] Marshall J.S., Palmer W.M. (1948). The distribution of raindrops with size. J. Meteor..

[82] Llort X., Sánchez-diezma R., Rodríguez A., Sancho D., Berenguer M., Sempere-torres D. Floodalert: A simplified radar-based ews for urban flood warning. Proceedings of the 11th International Conference on Hydroinformatics HIC.

[83] World Meteorological Organization (WMO) (2014). Guide to Meteorological Instruments and Methods of Observation: (CIMO Guide).

[84] Colom J.G., Cruz-Pol S., Pablos G., Trabal J.M. (2010). UPRM Weather Radars at the Central American and Caribbean Games at Mayagüez. IEEE Geosci. Remote Sens. Soc. Newsl..

[85] Texas Instruments (2011). Liquid-Level Monitoring Using a Pressure Sensor. http://www.ti.com/lit/an/snaa127/snaa127.pdf.

[86] Flow Line Options Corp (2010). Ultrasonic Transmitters vs. Guided Wave Radar for Level Measurement..

[87] Koval L., Vaňuš J., Bilík P. (2016). Distance Measuring by Ultrasonic Sensor. IFAC (International Federation of Automatic Control).

[88] Panda K.G., Agrawal D., Nshimiyimana A., Hossain A. (2016). Effects of environment on accuracy of ultrasonic sensor operates in millimetre range. Perspect. Sci..

[89] Stănescu T., Moldovan E.C., Dolga V. (2014). Effects of the Environment Temperature on the Characteristic of Parallax PING Ultrasonic Sensor. Robot. Manag..

[90] Saad C., Mostafa B., Cheikh E.A., Abderrahmane H. (2014). Comparative Performance Analysis of Wireless Communication Protocols for Intelligent Sensors and Their Applications. Int. J. Adv. Comput. Sci. Appl..

[91] Lee J.-S., Su Y.-W., Shen C.-C. A Comparative Study of Wireless Protocols: Bluetooth, UWB, ZigBee, and Wi-Fi. Proceedings of the IECON 2007 33rd Annual Conference of the IEEE Industrial Electronics Society.

[92] Vrije Universiteit Brussel—Department of Hydrology and Hydraulic Engineering (2017). FloodCitiSense: Early Warning Service for Urban Pluvial Floods for and by Citizens and City Authorities. http://www.iiasa.ac.at/web/home/research/researchPrograms/EcosystemsServicesandManagement/FloodCitiSense.html.

[93] Parker D.J. (2017). Flood Warning Systems and Their Performance. Oxford Research Encyclopedia of Natural Hazard Science.

[94] Rapant P., Inspektor T. Early warning of flash floods based on the weather radar. Proceedings of the 2015 16th International Carpathian Control Conference (ICCC).

